# Pea protein preload improves postprandial glucose response in healthy adults: a randomized, double-blind, controlled pilot study

**DOI:** 10.1007/s00394-026-03971-3

**Published:** 2026-06-11

**Authors:** Arig Elbira, Priscilla Grace Gnanasekaran Punithalayal, Alan Javier Hernández-Álvarez, Christine Boesch

**Affiliations:** 1 School of Food Science and Nutrition, Faculty of Environment, University of Leeds, Leeds, LS2 9JT UK; 2 National Alternative Protein Innovation Centre (NAPIC), Leeds, UK

**Keywords:** Pea protein, Meal timing, Glucose, Satiety, Blood pressure

## Abstract

**Purpose:**

Protein intake has shown benefits to mitigate postprandial hyperglycaemic excursions. In particular, whey protein has demonstrated strong potential for postprandial glucose management, and more recent findings highlighted evidence for increased efficacy of whey protein when consumed before, rather than with a carbohydrate-rich meal. Given the strong interest yet limited evidence on plant-based protein, the present study compared the potential of pea protein consumed prior to carbohydrates, on postprandial glucose as well as satiety and blood pressure (BP).

**Methods:**

In an acute randomized cross-over trial, ten healthy adults consumed a pea protein drink either before (PrePP) or with (PP) a standard carbohydrate-rich meal, compared with a control meal (CHO). Continuous glucose monitoring, satiety levels and BP were recorded over 180 min post-meal consumption.

**Results:**

Both PP and PrePP significantly reduced postprandial glucose excursion (0.46 vs. 1.125 mmol/L), compared to CHO (1.89 mmol/L). The effect was more pronounced with PrePP, exhibiting a delayed glucose response and a blunted peak beyond 60 min. Systolic BP remained unchanged, whereas both PP and PrePP significantly reduced diastolic BP compared to CHO (− 4.2 mmHg at 150 min, *p* < 0.05; −9.2 mmHg at 180 min, *p* < 0.01, respectively). PP significantly increased fullness and reduced hunger after 60 and 180 min, respectively, whereas PrePP significantly decreased hunger after 60 min of carbohydrate consumption.

**Conclusion:**

Given the enhanced efficacy in lowering postprandial glucose when consumed before carbohydrates, pea protein pre-meal consumption could be considered as part of a dietary strategy to manage postprandial glycaemia well comparable to whey protein.

**Supplementary Information:**

The online version contains supplementary material available at 10.1007/s00394-026-03971-3.

## Introduction

The increasing prevalence of metabolic disorders and cardiovascular diseases presents significant challenges to global health [1–3], highlighting the need for cost-effective dietary interventions e.g. to manage postprandial glycaemic excursions, such as hyperglycaemia— a key risk factor for cardiometabolic complications. Elevated postprandial glucose levels are consistently associated with increased cardiovascular risk, even in normoglycaemic individuals [4].

Since carbohydrates are the primary macronutrient influencing postprandial glycaemia, strategies that slow gastric emptying and modulate intestinal glucose absorption are of particular interest [5]. Protein intake has been widely explored as a dietary approach for improving glycaemic control [6, 7]. The mechanisms underlying the hypoglycaemic effects of proteins are multifactorial, involving enzymatic inhibition and hormonal regulation. Proteins can inhibit carbohydrate-digesting enzymes such as α-amylase and α-glucosidase, thereby slowing carbohydrate digestion and reducing glucose absorption [8].

Additionally, protein ingestion stimulates incretin hormones—glucose-dependent insulinotropic polypeptide (GIP) and glucagon-like peptide-1 (GLP-1)—which enhance insulin secretion, suppress glucagon release, and improve insulin sensitivity [9]. Proteins also influence gastric emptying, a major determinant of postprandial glucose response. This is partly mediated by leptin release from gastric chief cells, acting via CCK1 receptors to reduce gastric motility and increase satiety [10].

Importantly, the timing of protein intake influences its metabolic effects. Pre-meal protein consumption appears more effective than co-ingestion in attenuating postprandial glycaemia. A systematic review and meta-analysis by Smedegaard et al. found that whey protein preloads significantly reduced peak glucose (− 0.62 mmol/L) and 2-hour iAUC (− 39.8 mmol·min/L), particularly with doses ≥ 20 g taken 15–30 min before a carbohydrate-rich meal [11].

This enhanced effect is likely due to the time required for protein digestion and the early release of plasma amino acids and incretin hormones, which prepare the body for incoming glucose [12]. In contrast, simultaneous intake of protein and carbohydrate may not allow sufficient time for these mechanisms to take effect [13]. These findings underscore the relevance of meal sequencing and suggest that the strategic timing of protein intake may enhance its clinical utility in managing postprandial hyperglycaemia.

Supporting this, in vitro studies showed that protein digestion can generate bioactive peptides targeting glucose metabolism [14]. For example, the milk-derived peptide Ile–Pro–Ile (IPI) inhibits DPP-IV (IC₅₀ ~5 µM), potentially enhancing incretin activity [15]. A recent study identified 14 peptides from pea protein with α-amylase inhibitory properties, highlighting its potential to reduce carbohydrate breakdown [16].

Emerging evidence suggests that dietary proteins also influence blood pressure. An umbrella review of 16 systematic reviews found that higher protein intake, especially from milk-derived sources, was associated with modest reductions in systolic and diastolic blood pressure [17]. These hypotensive effects may involve multiple mechanisms. Arginine-rich proteins enhance nitric oxide production via eNOS, promoting vasodilation and reducing vascular resistance [18]. Protein-derived peptides may also inhibit angiotensin-converting enzyme (ACE), thereby reducing the formation of the vasoconstrictor angiotensin II. Moreover, protein-induced improvements in insulin sensitivity can indirectly lower blood pressure by counteracting hyperinsulinaemia, a known contributor to hypertension [19].

While most research has focused on animal proteins like whey, interest in plant-based proteins is growing due to their affordability, lower allergenicity, and environmental sustainability. Among these, pea protein stands out for its favourable amino acid profile and low glycaemic index [20]. Pea protein has been associated with benefits in weight regulation, gut health, and cardiometabolic outcomes. Several studies have shown that co-consumption of pea protein with carbohydrate-rich meals can modestly reduce postprandial glucose and enhance satiety [21–23]. However, the effects of consuming pea protein prior to a carbohydrate-rich meal remain under-investigated.

Therefore, the present study aimed to investigate the effect of pea protein consumption—either before or with a carbohydrate meal—on postprandial glucose response in healthy adults. Secondary outcomes included subjective appetite ratings and blood pressure responses. This study adds to the limited literature on the timing of plant protein intake and its potential role in managing postprandial metabolic markers.

## Materials and methods

### Study design

This study was conducted as a randomized, double blind, controlled, crossover trial at the Human Study Facility in the School of Food Science and Nutrition, University of Leeds, UK. A sample size of 10 participants was considered appropriate based on evidence from previous acute nutritional intervention studies, which have shown that this number is typically sufficient to detect a 1 mmol/L difference in postprandial glucose peak response [24, 25]. Ethical approval for the study was granted by the University of Leeds, Faculty Ethics Committee, AREA FREC 2023-0725-778. All procedures were conducted in accordance with the Declaration of Helsinki guidelines. Written informed consent was obtained from all participants prior to enrolment.

Participants were recruited between May and July 2024, and each participant attended three separate sessions over a two-week period, with a minimum of two days between sessions to allow for washout [26]. Participants were recruited for the postprandial intervention study via flyers and email distributed to staff and students. The recruitment period spanned several weeks, during which potential participants were invited to complete an initial phone/email questionnaire that collected demographic data, medical history, and lifestyle information.

Eligibility criteria included healthy adults, aged 18–65 years, within normal range of fasting blood glucose levels and body mass index (BMI)². The exclusion criteria were as follows: obesity (BMI ≥ 30 kg/m²), elevated fasting blood glucose (above 5.5 mmol/L), pregnancy, smoking, and any disease, allergies, or medication use known to affect food digestion, appetite, food sensory perception, or glucose metabolism. Those individuals who met the inclusion criteria were subsequently contacted for a screening visit, held one to two days before the first study session, to confirm eligibility and discuss study requirements in greater detail. Participants were asked to attend the screening visit after an overnight fast. During the visit, height and weight were measured to determine BMI; and a Continuous Glucose Monitor (CGM; FreeStyle LibrePro, Abbott, Wiesbaden, Germany) was fitted on the upper arm. Fasting glucose values from the CGM were reviewed to confirm participants were within the normal range prior to participation. The CGM then remained in place for the entire two-week period with interstitial glucose being recorded every 15 min.

### Study procedure

The study compared three conditions: a carbohydrate meal consumed together with a pea protein drink (PP); a pea protein drink consumed 30 min prior to the carbohydrate meal (PrePP); and a carbohydrate meal consumed alone as a control (CHO). The order of interventions was randomized using pre-generated sequences from an online program (http://www.randomization.com). Each session commenced at 9 am following a 10–12 h overnight fast. Participants were instructed to avoid high-protein meals, alcohol, and vigorous exercise for at least 24 h prior to each session and were asked to record their dietary intake during the preceding 24 h.

### Study interventions

In each intervention session, participants consumed 30 g of Pea Protein powder (Pulsin Ltd, UK) mixed with 230 mL of nitrate-free water (Buxton, Nestlé), alongside 109 g of Warburtons White Bread, which provides 50 g available carbohydrates. According to manufacturer information, a 30 g serving of Pulsin pea protein provides approximately 112 kcal, 24 g of protein, 1.6 g of carbohydrates, and 1.8 g of fat. To improve palatability and ensure sensory consistency across conditions, Ribena Blackcurrant Cordial (70 mL) was added to all drinks. This cordial is zero sugar and calorie-free and was used solely to mask the flavour of the protein and ensure all drinks appeared and tasted similar.

To maintain double-blind conditions, all drinks were prepared by a separate researcher not involved in data collection. Drinks were served in black opaque cups with lids to prevent visual identification of the beverage contents.

### Study measurements

On each study day, participants were seated in an armchair in an upright position and asked to rest quietly for at least 15 min in preparation for baseline measurements. The CGM sensor was scanned using the reader to obtain the baseline glucose value, and baseline blood pressure and visual analogue scale (VAS) ratings for satiety were also recorded. Blood pressure was measured three times at 3-min intervals using an automatic device (DINAMAP ProCare 100; GE Medical Systems, Milwaukee, WI, USA), with repeat measurements performed in triplicate. After these baseline assessments, participants consumed the designated meal or drink according to their randomized condition. Time zero (T0) was defined as the point when the carbohydrate meal was consumed. In the preload condition, the pea protein drink was consumed at -30 min (T-30), and the subsequent time points for measurement were based on this reference. Postprandial responses were monitored over a three-hour period, during which systolic and diastolic blood pressure were measured every 30 min. Additionally, subjective appetite levels were recorded using a 100 mm paper-based VAS at same intervals. Participants were asked to indicate their feelings of hunger, fullness, desire to eat and prospective food intake by marking a point on a horizontal 100 mm line, where one end represented “not at all” and the other “extremely” for each sensation. Continuous glucose monitoring via CGM provided detailed data on postprandial glucose responses throughout the study.

### Statistical analysis

The primary objective of this trial was to compare postprandial interstitial glucose responses, measured via continuous glucose monitoring, when pea protein was consumed either prior to or together with a carbohydrate meal, compared to a carbohydrate-only control. Secondary outcomes included blood pressure and subjective appetite ratings.

The effect of the three interventions on peak postprandial interstitial glucose rise (C-max), was assessed using a two-factor repeated measures ANOVA. Where significant effects were found, post hoc comparisons were conducted using Tukey’s test. Postprandial interstitial glucose responses were expressed as delta values from baseline, and incremental area under the curve (iAUC) values were calculated using the trapezoidal rule over 60, 90, 120, and 180 min following the carbohydrate meal [27, 28]. The trapezoidal rule estimates the area under the curve by dividing it into a series of adjacent trapezoids formed between each pair of time points and summing their areas, excluding values falling below baseline. These iAUC values were analysed using one-way ANOVA. For outcomes where values below baseline were relevant—such as blood pressure and subjective appetite ratings—total area under the curve (tAUC) was calculated instead of iAUC. Blood pressure, as well as subjective ratings of hunger, fullness, and prospective food intake, were analysed using one-way ANOVA alongside their tAUC values. Where significant differences were detected, post hoc comparisons were conducted using Tukey’s test.

All statistical analyses were performed using GraphPad Prism (version 10.2.0) with *p* < 0.05 considered as significantly different. Data are reported as mean values ± standard error of the mean (SEM).

## Results

**Fig. 1:**
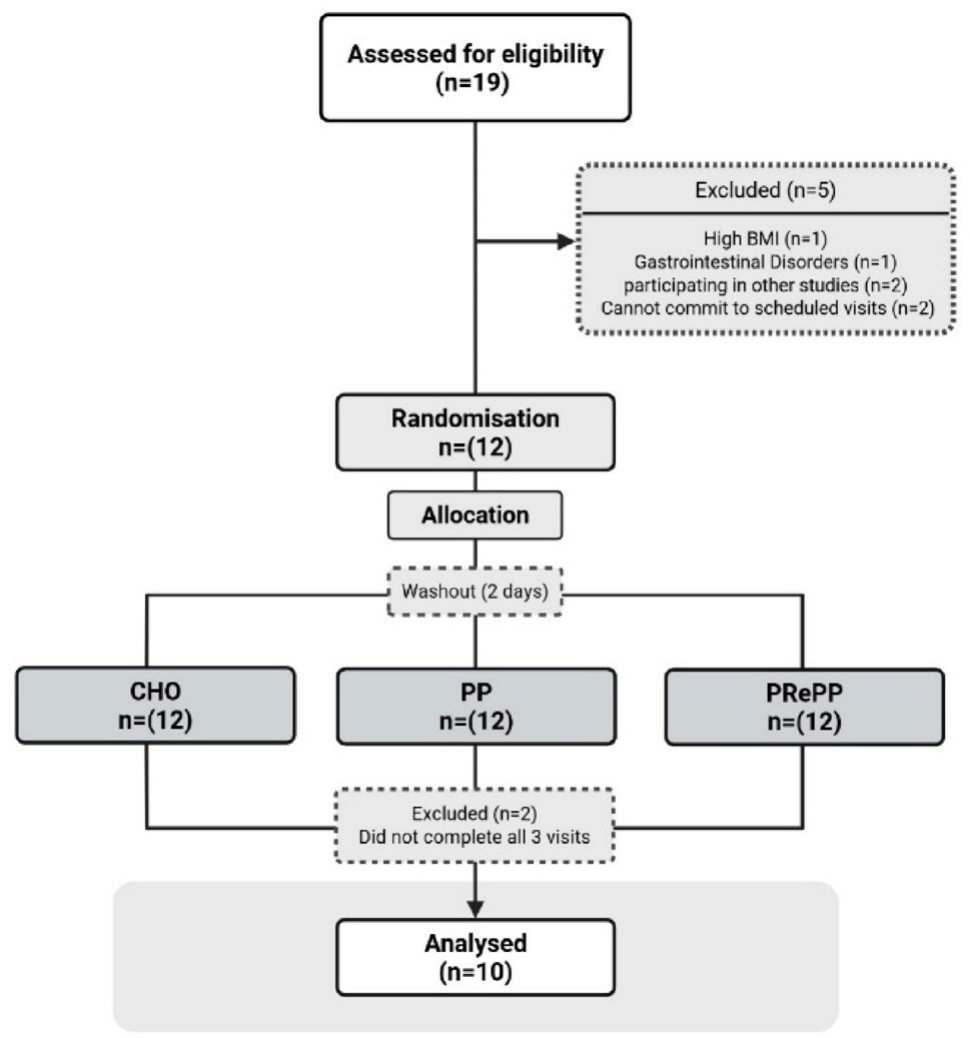
A flow diagram of the participant recruitment

A total of 19 individuals were initially screened for eligibility to participate in the trial. Of these, 14 volunteers attended the screening visit, and 10 participants (8 females and 2 males) successfully completed all three study visits (Fig. 1). Detailed baseline characteristics of the study participants are provided in Table 1.

**Table 1:** Baseline characteristics of study participants

	Mean	SEM
Age (y)	25.6	6.6
Height (cm)	166.5	3.4
Weight (kg)	63.1	3.5
Body mass index (kg/m2)	22.7	0.3
Fasting glucose (mmol/L)^a^	5.1	0.2
Systolic Blood Pressure (mmHg)	118	8.1
Diastolic Blood Pressure (mmHg)	69.3	5.8

^a^measured by Continuous Glucose Monitors.

### Postprandial interstitial glycaemic responses

All participants presented with fasting interstitial glucose values below 5.5 mmol/L on all study visits, with no significant differences in baseline interstitial glucose values between the intervention arms. Additionally, no effects of gender, age, or BMI were observed on the fasting interstitial glucose levels.

**Fig. 2:**
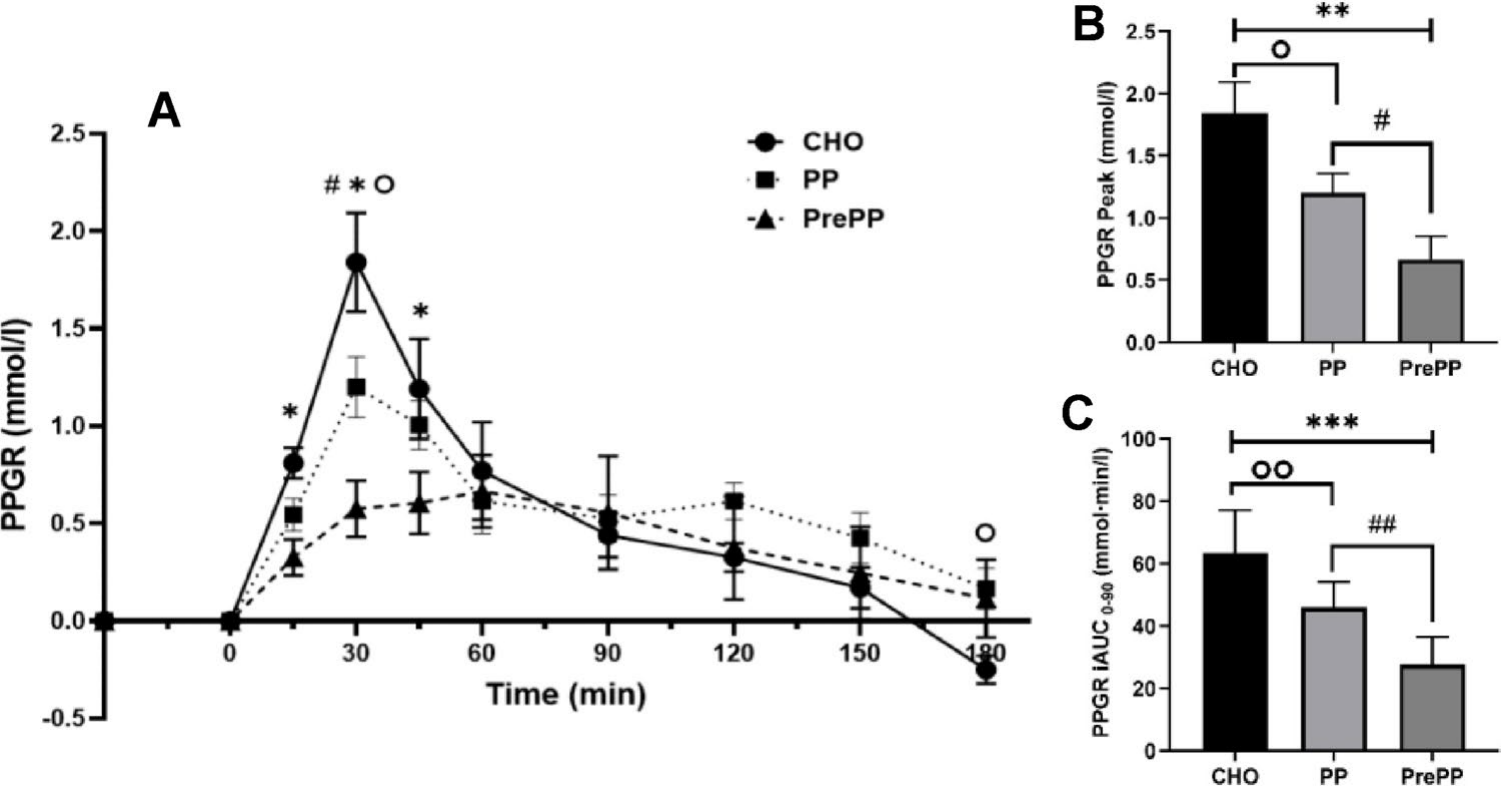
Incremental blood glucose response over 180 min following consumption of a carbohydrate meal with a control drink CHO (●), pea protein drink consumed simultaneously (PP) (■) or as a preload (PrePP) (▲) (**A**); as well as peak (C-max) glucose (**B**) and iAUC0-60 (**C**). Data are presented as mean ± SEM for 10 participants. Post hoc comparisons of time-point changes from baseline were performed using Tukey’s adjustment (*P* < 0.05), assessing differences between CHO vs. PP, CHO vs. PrePP, and PP vs. PrePP. *, # and ° indicate significant differences between CHO and PrePP, PP and PrePP and PP and CHO, respectively

Postprandial glucose response (PPGR) was significantly reduced in both the PP and PrePP groups compared to the CHO group (*p* < 0.001). Specifically, the reduction was more pronounced when protein was consumed as a preload PrePP (C-max: 0.7 ± 0.5 mmol/L), which was significantly lower than both the PP (C-max: 1.2 ± 0.7 mmol/L) and CHO (C-max: 1.85 ± 0.8 mmol/L) groups (*p* < 0.001) (Fig. 2a). Additionally, the PrePP group showed a delayed time to peak, occurring at 60 min post-consumption, while both the PP and CHO groups reached peak glucose at 30 min (Fig. 2b). The mean glucose iAUCs (0–180 min) were lower in the PrePP group compared to both the PP and CHO control groups, although this difference did not reach statistical significance (Fig. 1c). A significant time × intervention interaction was observed when analysing postprandial interstitial glucose concentrations in response to the test meals (*p* < 0.001).

### Postprandial blood pressure responses

**Fig. 3:**
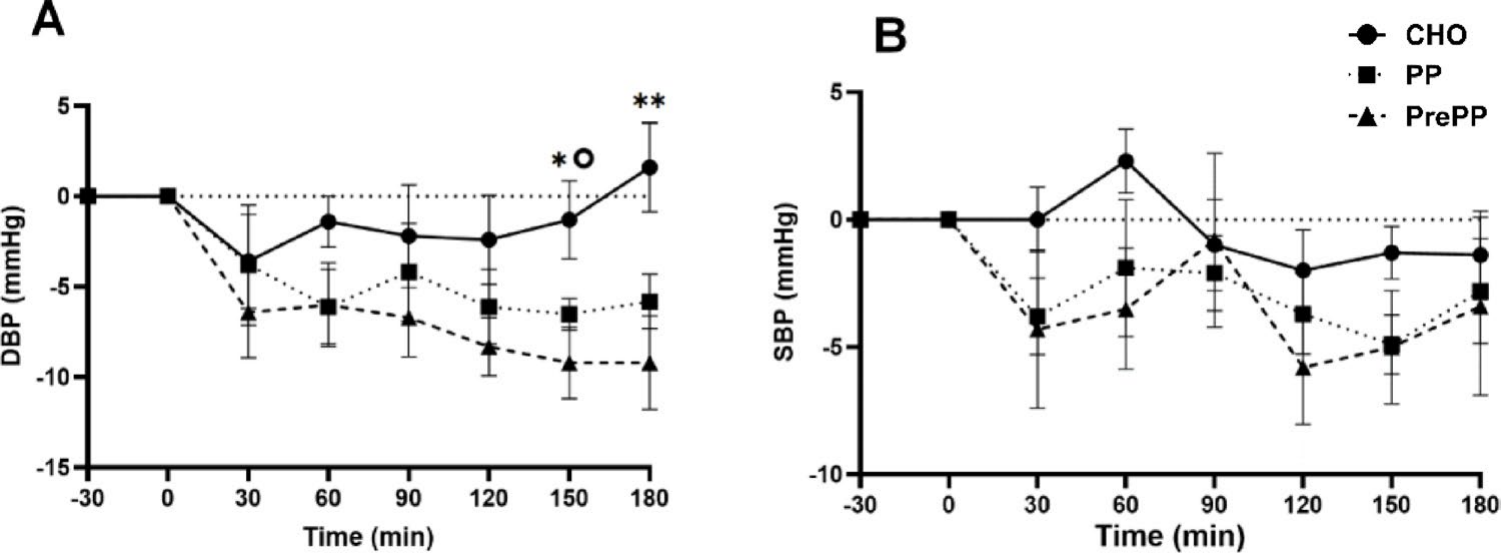
Mean change from baseline in diastolic blood pressure (DBP) (**A**) and systolic blood pressure (SBP) (**B**) over 180 min following consumption of a carbohydrate meal with a control drink CHO (●), pea protein drink consumed simultaneously (PP) (■) or as a preload (PrePP) (▲); Data are presented as mean ± SEM for 10 participants. Post hoc comparisons of time-point changes from baseline were performed using Tukey’s adjustment (*P* < 0.05), assessing differences between CHO vs. PP, CHO vs. PrePP, and PP vs. PrePP. The symbols * indicate a significant difference between CHO and PrePP, ° denotes a significant difference between PP and CHO

Diastolic blood pressure (DBP) significantly decreased following both protein interventions compared to the carbohydrate control as shown in Fig. 3a. The largest reduction was observed with PrePP, where DBP dropped by 9.2 mmHg at 180 min (*p* < 0.01), while PP showed a 4.2 mmHg decrease at 150 min (*p* < 0.05). In contrast, systolic blood pressure (SBP) did not exhibit statistically significant changes across the protein interventions. However, a notable early reduction in SBP was observed between 0 and 30 min, with decreases of 3.8 (*p* = 0.2) mmHg for PP and 4.3 mmHg for PrePP (*p* = 0.3), whereas the carbohydrate control (CHO) showed no change from baseline during this period (Fig. 3b).

### Subjective appetite responses

Table 2 presents the average subjective appetite ratings for all participants. No significant differences were observed between intervention groups in baseline measures of hunger, fullness, or prospective food intake (*p* > 0.05).

**Table 2:** Incremental subjective appetite responses as measured by Visual Analogue Scale over 180 min following intake of pea protein prior (PrePP) or simultaneously (PP) with a carbohydrate-rich meal

	CHO^c^		PP		PrePP	
	Mean	SEM	Mean	SEM	Mean	SEM
Hunger score 60 min	23.8^a^	4.7	21.9^a^	2.9	15.3^b^	3.0
Hunger score 180 min	39.5^a^	6.3	29.6^b^	3.7	33.2 ^a^	4.8
Hunger AUC_0–180_ (mm h)	97.3	13.2	87.8	10.0	89.6	9.8
Fullness score 60 min	46.1^a^	5.8	56.1^b^	4.6	51.8^a^	4.9
Fullness score 180 min	33.5	6.0	33.8	4.1	31.8	5.8
Fullness total AUC_0–180_ (mm h)	105	14.6	109	7.9	103	9.2
PFI score 60 min	35.2	5.4	33.8	4.4	34.1	5.2
PFI score 180 min	42.5	6.0	41.2	5.1	43.0	5.1
PFI AUC_0–180_ (mm h)	110.7	13.1	107.5	11.0	109.8	12.9

CHO, control drink; PP, pea protein drink consumed simultaneously; PrePP, pea protein drink consumed as preload; PFI, prospective food intake

^c^*n* = 10

Different superscript letters within a row indicate significant differences (Tukey’s post hoc test, *p* < 0.05)

Subjective ratings of fullness at 60 min were significantly higher following PP compared to the control (*p* < 0.05), while hunger ratings were significantly lower in PP versus control at 180 min (*p* < 0.05). As well, PrePP resulted in significantly reduced hunger at 60 min post-consumption compared to the control (*p* < 0.05). No significant differences were observed between conditions for prospective food intake during the postprandial period. However, subgroup analysis revealed significantly lower hunger ratings at 60 min and tAUC 0–3 h following PrePP consumption in individuals who typically consume a light breakfast (*n* = 6), compared to those who habitually eat a heavier breakfast (*n* = 4). There were no significant gender × intervention interactions for hunger, fullness, or prospective food intake *(p* > 0.05); however, these findings should be interpreted with caution given the small number of male participants (*n* = 2).

## Discussion

The present study aimed to examine the impact of the timing of pea protein consumption on postprandial interstitial glucose response. The findings revealed that consuming pea protein as a preload resulted in a more pronounced reduction in postprandial glucose peak compared to when it was consumed with the meal. These results align with recent studies supporting the hypothesis that the ability of protein to modulate postprandial glucose levels is influenced by the timing of consumption, with greater effects observed when consumed before a meal [29–31] rather than during or after [32] the meal. For example, whey protein preloads have been shown to lower postprandial glucose AUC by 28–50%. Previous studies have shown that a single dose of 50 g whey protein or a 4-week regimen of 25 g whey protein taken three times daily improved postprandial hyperglycaemia when consumed before a mixed meal [33].

The observed reduction in postprandial glucose excursion in the protein preload group (PrePP) can be attributed to multiple mechanisms related to protein digestion kinetics, hormonal responses, and enzyme inhibition [5]. Unlike the PP group, where protein was consumed alongside carbohydrates, pre-consumption of protein likely allows for its rapid digestion before carbohydrate ingestion. This early digestion may facilitate several metabolic effects that contribute to glycaemic control, including inhibition of carbohydrate digestion through enzymatic suppression, impact on early-phase insulin secretion and modulation of incretin hormones [11, 12].

One of the primary mechanisms through which the pea protein preload reduces postprandial glucose levels is by inhibiting key carbohydrate-digesting enzymes, particularly α-amylase and α-glucosidases. These enzymes are responsible for hydrolysis of dietary starch into glucose, which can then be absorbed. The timing of protein ingestion plays a critical role in modulating postprandial glycaemic responses [31, 33]. Consuming protein before carbohydrate intake allows for early protein digestion and absorption of amino acids and bioactive peptides, which can subsequently exert inhibitory effects on digestive enzymes once carbohydrates reach the small intestine. This delayed carbohydrate digestion results in a slower and more sustained glucose release into circulation, preventing sharp postprandial spikes [5, 22]. Peptides released during pea protein hydrolysis can interact with the active site of α-amylase, competitively inhibiting its function [8, 16]. The glucose lowering mechanisms have been well-documented in in vitro studies investigating plant-derived peptides, where hydrolysed protein fractions exhibit significant α-amylase inhibitory activity [14]. For example, Mojica et al. demonstrated that peptides from legume protein hydrolysates, including those from pea and common bean, effectively inhibited α-amylase activity in vitro, leading to a reduction in carbohydrate digestion and postprandial glucose levels [34].

Another potential mechanism involves the stimulation of gut hormones that regulate glucose metabolism. Protein preload has been shown to enhance the secretion of glucagon-like peptide-1 (GLP-1) and gastric inhibitory polypeptide (GIP), which promote insulin release and suppress postprandial glucose excursions [29, 35]. Additionally, GLP-1 slows gastric emptying, further contributing to delayed glucose absorption. Previous human studies on whey protein have evidenced that the pre-meal consumption of protein can indeed enhance early insulin release resulting in improved glycaemic control [31, 36, 37]. A more rapid protein digestion and absorption of protein-derived amino acids and peptides leads to direct and/or early stimulation of pancreatic β-cells, enhancing insulin release [38, 39]. For instance, amino acids such as leucine, lysine, and arginine, are known to be potent insulin secretagogues [40]. While our study did not measure insulin and other gut hormones, the delayed peak glucose concentration in the preload group (60 min compared to 30 min in other 2 groups) suggests a potential early-phase insulin secretion-mediated effect, as has been observed in previous whey protein preload study [35]. Future studies should investigate whether pea protein preload stimulates postprandial hormone secretion i.e. insulin, PYY and GLP-1, similarly to whey protein.

A previous study that compared whey and pea protein preloads, has shown that whey protein tends to have a more pronounced effect in reducing postprandial glucose levels; however, the control meal in that study was offered ad libitum rather than as standardised carbohydrate intake which was used in the present study [41]. The absence of a standardized meal could introduce variability that may influence the results. Thus, caution should be exercised when interpreting the outcomes of such studies, as the type and amount of carbohydrate consumed may significantly affect the postprandial response.

Another key finding of our study was that pea protein consumption significantly reduced DBP, with the most pronounced effect occurring between 150 and 180 min postprandially. These results suggest that pea protein itself is the primary driver of the blood pressure-lowering effect, regardless of timing, although consuming it prior to the carbohydrate meal appeared to produce a slightly more pronounced response. While there is no direct evidence showing that protein preload is more effective than co-consumption in reducing blood pressure, several studies on whey protein provide valuable insights [42]. Whey protein, a rapidly digestible animal protein, has demonstrated significant blood pressure-lowering effects primarily through its bioactive peptides, such as angiotensin-converting enzyme (ACE) inhibitors [43]. These peptides inhibit the conversion of angiotensin I to angiotensin II, a potent vasoconstrictor, thereby promoting vasodilation and reducing blood pressure. Whey protein rapid gastric emptying and efficient enzymatic hydrolysis in the stomach and small intestine enable the early release and absorption of these peptides, potentially allowing its effects to manifest even shortly after ingestion.

In contrast, plant proteins like pea protein are less soluble and structurally more complex, making them slower to digest compared to whey [44]. Pea protein consists predominantly of globulins—mainly legumin and vicilin—which possess tightly folded tertiary and quaternary structures stabilized by disulfide bonds, rendering them more resistant to enzymatic hydrolysis. In comparison, whey protein contains more readily digestible, loosely folded proteins such as β-lactoglobulin and α-lactalbumin, which facilitate faster breakdown and absorption. As a result, the release of bioactive peptides from pea protein may be delayed, requiring more time to undergo enzymatic hydrolysis and absorption. For example, in vitro studies have demonstrated significant ACE inhibitory activity of hydrolysed pea protein, suggesting that its peptides can contribute to blood pressure regulation when sufficiently digested [45, 46]. In our study, the 30-min timeframe likely provided sufficient time for the digestion and absorption of these peptides, maximizing their potential to lower blood pressure before the carbohydrates reached the small intestine. This timing of consumption likely enhanced the efficacy of pea protein, explaining the observed significant reduction in 150 min postprandial blood pressure when pea protein was consumed as a starter compared to co-consumption.

Regarding the satiety response, both protein interventions significantly influenced aspects of appetite, reflected by higher subjective fullness scores and lower hunger ratings following pea protein ingestion compared to the carbohydrate control. Although whole peas are rich in dietary fibre, which can contribute to feelings of fullness [20], the pea protein product used in this study contained negligible fibre; therefore, the observed effects are unlikely to be fibre related [47, 48]. However, this effect was time-specific and did not translate into significant differences in overall appetite measures such as prospective food intake or total appetite tAUCs. The results align with previous research showing limited or inconsistent satiety responses to protein preloads [49].

A limitation of our study is the high inter-individual variation in postprandial interstitial glucose responses, which may have masked potential differences in overall glucose iAUC between groups. Although, this finding is consistent with some studies using whey protein, where despite a significant reduction in peak glucose levels, the total glycaemic response over time remained unchanged [29, 31]. Several factors may explain this discrepancy. Individual differences in insulin sensitivity, glucose handling, and gut hormone responses could have influenced glycaemic outcomes. Variations in eating speed and salivary response may also have played a role. Another limitation is the lack of a vehicle-only preload control drink (no protein, matching for carbohydrate and fat), which would have further isolated the protein effect. However, as the preload provided only 1.6 g carbohydrate and 1.8 g fat, it is unlikely to have meaningfully influenced glucose or blood pressure responses [50]. The within-subject design also minimized any potential vehicle-related bias.

A further important consideration is the relevance of changes in blood pressure determined in acute settings. The observed reduction in DBP following pea protein ingestion reflects short-term physiological responses, indicating potential for long-term hypotensive effects. Longer-term intervention studies are needed to determine whether chronic consumption would translate into chronic benefits to lower blood pressure. Nevertheless, similar acute postprandial BP reductions after protein ingestion have been reported [51] and longer-term protein interventions show modest but significant BP reductions when sustained [52, 53].

Lastly, although the current pilot study provides highly valuable data [25, 26], a larger and more sex balanced sample size should be preferred for future studies to reassure differences in overall glucose response and other outcomes. Existing evidence suggests that in healthy adults under normal physiological conditions, these influences are relatively modest, and both sexes may show similar postprandial glucose and insulin responses when matched for age, BMI, and energy intake [54].

These considerations also highlight the need for further studies to explore the mechanisms behind glucose-lowering effects of proteins, e.g. to impact postprandial hormone profiles.

In conclusion, this pilot study indicates that pea protein preload can effectively reduce postprandial blood glucose levels and may also assist in managing blood pressure. While much of the research has focused on whey protein, our findings suggest that pea protein may offer similar benefits in reducing postprandial glucose excursions. Given the increasing demand for plant protein and high protein ingredients, the current results demonstrate promising potential for these to be applied in the context of glucose management and hypertension. However, further research is needed to better understand the mechanisms underlying these effects and to determine the efficacy of longer-term consumption to improve metabolic outcomes. Additionally, future work should explore the glycaemic and metabolic effects of other plant and alternative protein sources to expand the range of dietary protein for effective blood glucose and blood pressure management.

## Supplementary Information

Below is the link to the electronic supplementary material.

Supplementary Material 1

## Data Availability

Anonymised data are available from the corresponding author upon reasonable request.
